# Diagnostic accuracy of a urine dipstick for detecting albuminuria in hypertensive patients

**DOI:** 10.12688/f1000research.25564.2

**Published:** 2021-07-07

**Authors:** Phornwipa Panta, Win Techakehakij

**Affiliations:** 1 Department of Social Medicine, Lampang Hospital, Amphur Muang, Lampang, Thailand

**Keywords:** hypertension, albuminuria, urine dipstick, diagnostic test

## Abstract

**Background:** Screening for albuminuria is generally recommended among patients with hypertension. While the urine dipstick is commonly used for screening urine albumin, there is little evidence about its diagnostic accuracy among these patients in Thailand. This study aimed to assess the diagnostic accuracy of a dipstick in Thai hypertensive patients for detecting albuminuria.

**Methods:** This study collected the data of 3,067 hypertensive patients, with the results of urine dipstick and urine albumin-to-creatinine ratio (ACR) from random single spot urine being examined in the same day at least once, at Lampang Hospital, Thailand, during 2018. For ACR, a reference standard of ≥ 30 mg/g was applied to indicate the presence of albuminuria.

**Results:** The sensitivity, specificity, positive predictive value (PPV), and negative predictive value of the trace result from dipsticks were 53.6%, 94.5%, 86.5%, and 75.5%, respectively. The area under the receiver operating characteristic curve of the dipstick was 0.748.

**Conclusion:** Using the dipstick for screening albuminuria among hypertensive patients should not be recommended for mass screening due to its low sensitivity. In response to high PPV, a trace threshold of the dipstick may be used to indicate presence of albuminuria.

## Introduction

Strong evidence has indicated that the presence of albuminuria in hypertensive patients is associated with the development of chronic kidney disease (CKD), which increases the risk of cardiovascular-related morbidity and mortality ^1, 2^. Early detection of CKD is important as either angiotensin-converting-enzyme inhibitor drugs or angiotensin II receptor blocker drugs can be added to a patient’s treatment regimen to slow down the progress of the disease and thus reduce all-cause mortality.

Detection of albumin in urine plays an important role in diagnosing CKD in the early stages. Regarding the detection of albumin in urine, urine albumin-to-creatinine ratio (ACR) has widely been recommended to be used in diagnosing albuminuria, which is defined as the amount of urine albumin divided by urine creatinine ≥ 30 mg/g [≥ 3 mg/mmol] ^3– 5^.

Despite the recommendations, performing ACR in all patients with hypertension is not always applicable, particularly in a primary care unit in rural or outreach areas where the necessitated resources may be unavailable. Practically, the urine dipstick is a test that has widely been used to identify the presence of albumin in the urine due to its low cost and high accessibility.

Although using the urine dipstick is pragmatic, existing literature has not affirmed the accuracy of the test. Previous research has revealed a variety of diagnostic accuracy of the urine dipstick, compared with ACR. While some studies suggest that the dipstick is inappropriate for screening albuminuria ^6– 9^, others conclude that trace albuminuria from a dipstick can be used to indicate the presence of urine albumin ^10, 11^.

Owing to result inconsistencies, it is still arbitrary as to whether or not positive findings of albumin from a urine dipstick could be used to confirm presence of albuminuria. Additionally, there is as yet no evidence to demonstrate if diagnostic results would be consistent across populations. Therefore, this study aimed to assess the diagnostic accuracy of a dipstick in Thai hypertensive patients for detecting albuminuria.

## Methods

### Participants

This analysis is based on retrospective data from patients who visited Lampang Hospital from January to December 2018. The study included patients aged 18 years and over who were diagnosed with hypertension, ICD10 code “I10-14”, with the results of urine dipstick and ACR from random single spot urine being examined in the same day at least once. Laboratory results from the last visit were used if multiple results of a urine dipstick and ACR on the same day were presented within the same patient. Patients with urinary tract infections were excluded from the study.

This study protocol was approved by the Ethics Committee at Lampang Hospital (No.79/62). Consent of the patients to use their data in the study was waived by the ethical committee due to the retrospective nature of the study.

### Reference standard and index test

ACR was a reference standard to indicate the level of urine albumin. Evaluation of ACR was performed at Lampang Hospital using the immunoturbidimetric essay by AU5800/DxC700AU. The result of ACR ≥30 mg/g indicates the presence of albuminuria ^12, 13^.

This study employed the urine dipstick, “URiSCAN 9 SG” and the analyzer “URiSCAN SUPER+”, as an index test. Interpretation of the results were based on the color changes on the indicator tetrabromophenol blue in the presence of urine albumin. A positive reaction is indicated by a color change to yellow or green, reflecting the albumin results of negative, trace, 1+, 2+,3+, and 4+.

### Covariates

Demographic characteristics including age and sex were collected for use in the analysis. Body mass index was calculated by weight in kilograms divided by squared height in centimeters ^14^. Glomerular filtration rate (GFR) was estimated using the formula eGFR = 141 × min(S _Cr_/κ, 1) ^α^ × max(S _Cr_ /κ, 1) ^-1.209^ × 0.993 ^Age^ × 1.018 [if female] × 1.159 [if Black] ^15^. Information about patients’ underlying disease of diabetes was obtained from the diagnosis in the hospital’s electronic medical record with ICD10 code “E10-14” ^16^.

### Statistical analysis

Chi-squared test and t-test were applied to explore the association between the presence of albuminuria from ACR and covariates, with a significance level of 0.05. Sensitivity, specificity, positive predictive value, and negative predictive value of the dipstick were calculated, with 95% confidence intervals. The area under the receiver operating characteristic curve was approximated to demonstrate the test performance ^17^. Subgroup analyses using the trace threshold of dipstick were performed to elucidate the diagnostic accuracy of the test among subgroups. Statistical analyses were performed using STATA version 13 ^18^.

## Results

**Figure 1.**
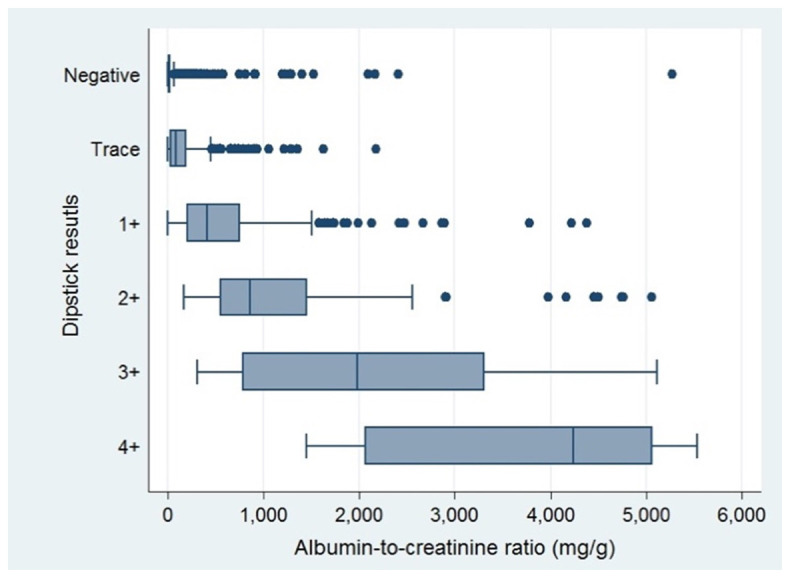
Distribution of albumin-creatinine ratios stratified by results of dipsticks.

 A total of 3,067 hypertensive patients matched the study criteria and were included in the analysis ( Table 1). Approximately 39.8% of the samples presented with albuminuria. The mean age of the patients was 63.7 year, with ~40% being men. Diabetes appeared among 73.7% of the patients; 17.7% of them had eGFR <60 ml/min/1.73m ^2^. Albuminuria was present in 24.5% of those with negative result from the dipsticks. Distribution of albumin-creatinine ratios with respect to results of urine dipsticks were exhibited in Figure 1.

**Table 1.** Demographic characteristics of the patients.

Characteristics	Albumin-to-creatinine ratio		P-value
	*<30 mg/g*	*≥ 30 mg/g*	
Total, n	1,847	1,220	
Gender, n (%)			
*Male*	736 (39.9)	484 (39.7)	0.937
*Female*	1,111 (60.2)	736 (60.3)	
Age years, mean±SD	63.52±10.3	64.0±10.7	0.238
Diabetes, n (%)	1,326 (71.8)	934 (76.6)	0.004
eGFR <60 mL/min/1.73 m2, n (%)	350 (19.0)	194 (15.9)	0.031
Urine albumin results from dipstick, n (%)			
*Negative*	1,745 (94.5)	566 (46.4)	<0.001
*Trace*	95 (5.1)	275 (22.6)	
*1+*	7 (.4)	226 (18.5)	
*2+*	0 (0.0)	120 (9.8)	
*3+*	0 (0.0)	25 (2.0)	
*4+*	0 (0.0)	8 (0.7)	
Body mass index, mean±SD	25.6±5.1	25.7±4.7	0.592

**Figure 2.**
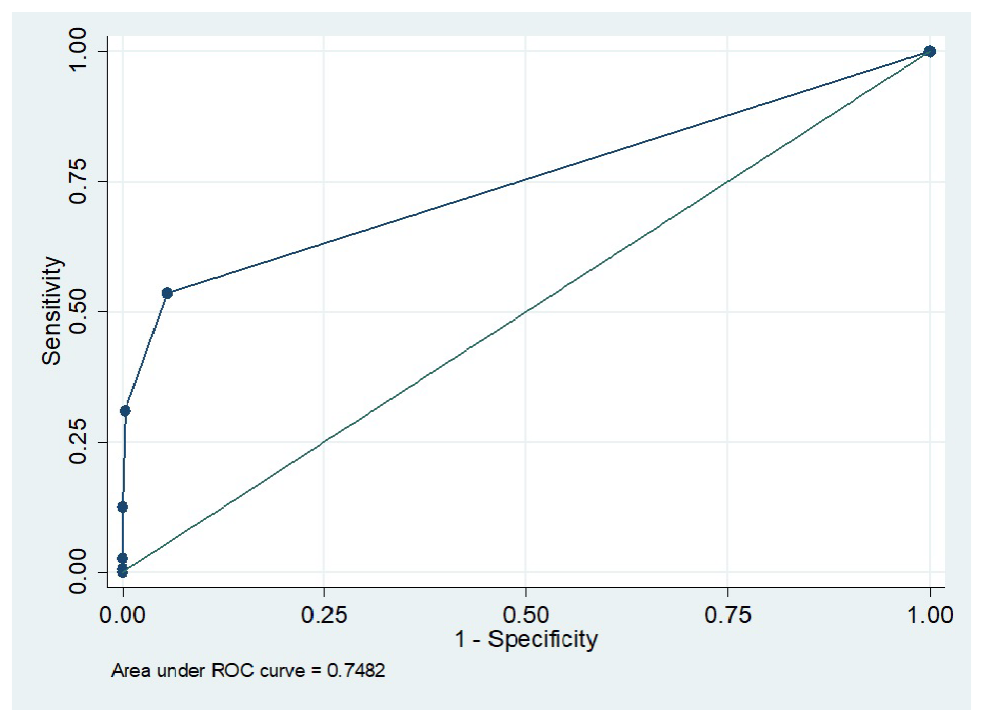
Receiver operating characteristic curve for the performance of dipsticks in detecting albuminuria.

Table 2 demonstrated the sensitivity, specificity, positive and negative predictive values of urine dipstick in detecting albuminuria. It is seen that sensitivity of 53.6% was observed when the trace threshold was applied, whereas cutoff of ≥2+ and higher yields 100% test specificity. The area under the receiver operating characteristic curve was 0.7482 ( Figure 2).

**Table 2.** Sensitivity, specificity, positive and negative predictive values of urine dipstick to detect albuminuria.

Cutoffs	Sensitivity (%)	Specificity (%)	Positive predictive value (%)	Negative predictive value (%)
**≥Negative**	100.0	0.0	39.8	
**≥Trace**	53.6	94.5	86.5	75.5
	95% CI 50.8-56.4	95% CI 93.3-95.5	95% CI 83.9-88.9	95% CI 73.7-77.3
**≥1**	31.1	99.6	98.2	68.6
	95% CI 28.5-33.1	95% CI 99.2-99.8	95% CI 96.3-99.3	95% CI 66.8-70.4
**≥2**	12.5	100.0	100.0	63.4
	95% CI 10.7-14.5	95% CI 99.8-100.0	95% CI 97.6-100.0	95% CI 61.6-65.1
**≥3**	2.7	100.0	100.0	60.9
	95% CI 1.9-3.8	95% CI 99.8-100.0	95% CI 89.4-100.0	95% CI 59.1-62.6
**≥4**	0.7	100.0	100.0	60.4
	95% CI 0.3-1.3	95% CI 99.8-100.0	95% CI 63.1-100.0	95% CI 58.6-62.1

CI, confidence interval.

Comparing diagnostic accuracy of the dipstick, it appears that sensitivity, specificity, along with positive and negative predictive values were approximately the same in all subgroups ( Table 3). 

**Table 3.** Diagnostic performance of the urine dipstick result of trace and higher for detection of albumin-to-creatinine ratio ≥ 30 mg/g.

Characteristics	Sensitivity (%)	Specificity (%)	PPV (%)	NPV (%)
All samples	53.6	94.5	86.5	75.5
Gender				
*Male*	54.6	94.2	86.0	75.9
*Female*	53.0	94.7	86.9	75.6
Age group, years				
*<60*	52.6	93.8	84.7	75.3
*≥60*	54.1	94.8	87.5	75.6
Diabetes				
*Yes*	54.2	94.3	87.1	74.5
*No*	51.8	94.8	84.6	78.1
eGFR category				
*≥60 mL/min/1.73 m2*	54.4	94.3	86.8	75.1
*<60 mL/min/1.73 m2*	49.5	95.1	85.0	77.3
Body mass index category				
*<23*	56.1	93.3	83.6	77.8
*≥23*	52.6	95.0	87.9	74.5

PPV, positive predictive value; NPV, negative predictive value

## Discussion

Existing studies have manifested a wide range of positive predictive values (PPVs) of urine dipsticks among patients with hypertension, ranging from 27 to 82 ^7, 19^. However, none have been conducted in a Thai population. Results of this study, exploring the diagnostic accuracy of the dipstick in a Thai population, not only illustrates the outcomes in this specific population, but can also be used in comparison with results from other populations for a better understanding of test accuracy.

Previous research has documented the differences in sensitivity and specificity of the dipstick across populations. A Japanese study showed sensitivity, specificity, and PPV of 37.1%, 97.3%, and 71.4%, respectively ^11^. Another study conducted in Australian adults showed sensitivity, specificity, and PPV of 69.4%, 86.8%, and 27.1%, respectively ^7^. One possible explanation for the difference in diagnostic accuracy of the dipstick was owing to differences in the characteristics of the populations ^7^. The other study points out variation in the calibration of the dipstick as another explanation for differences between populations ^9^. Compared with previous reports, diagnostic parameters shown in this study affirms variation in diagnostic performance of the dipstick across populations. This implies that the assessment of dipstick performance should be recommended for different populations.

It should be noted that false positive results of the dipstick could come from highly alkaline urine and contamination of antiseptics. Moreover, urine specimens used in this study came from random spot urine collection, which may be subjected to false positive results. Likewise, false negative results may have occurred due to excessive hydration before collecting the urine specimen, which leads to a decrease in concentration of urine albumin and subsequently a smaller chance of detecting albuminuria.

Such low sensitivity of 53% from the urine dipstick indicates that almost half of the patients with albuminuria cannot be identified using just the urine dipstick. It is also seen that among patients with a negative albumin result from the dipstick, albuminuria was found in nearly a quarter of them. This outcome well aligns with previous studies asserting low sensitivity of the dipstick in detecting albuminuria ^6, 9, 11^. Given strong evidence indicating the high probability of cases being undetected, using the dipstick alone should not be recommended for use in screening of albuminuria among hypertensive patients.

Results from the study revealed a rather high predictability of the dipstick in detecting urine albumin. Concerning the dipstick cutoffs, applying the trace threshold yields a PPV of 86.5%, compared with 98.2% and 100% using the 1+ and 2+ thresholds, respectively. Though a rather high chance of predicting albuminuria once hypertensive patients have these results of trace or higher from the dipstick, it should be borne in mind that albuminuria may be overly diagnosed with the application of the trace threshold, compared with using the higher cutoffs.

Although excellent PPV can be achieved when employing higher thresholds of the dipstick, drawbacks remain when the recommendation for using the high threshold is applied due to fewer patients being applicable. Considering the trade-off between PPV and applicability of the dipstick results, the trace threshold may be recommended for indicating the presence of albuminuria in hypertensive patients.

Even though the KDIGO guidelines ^3^ have recommended the use of ACR to indicate the presence of albuminuria, this is proven to be rather costly and not readily available in some regions. Limitations, regarding the availability and costs of ACR, may arise when considering the application of ACR for routine screening of hypertensive patients. Nonetheless, evidence has demonstrated a low sensitivity of urine dipsticks, which should not be recommended for screening albuminuria. Hence, ACR is deemed the option for screening albuminuria in the setting where resources are available.

## Conclusion

While existing evidence is controversial to whether the urine dipstick should be recommended for screening albuminuria in hypertensive patients, results from this study demonstrated that the dipstick has such low sensitivity in detecting albumin in urine in the Thai population. These results suggest that the urine dipstick not be recommended for screening urine albumin in patients with hypertension. In contrast, results of trace or higher yields high PPV, indicates a very high possibility of the presence of microalbuminuria.

## Data availability

### Underlying data

Figshare: Diagnostic Accuracy of a Urine Dipstick for Detecting Albuminuria in Hypertensive Patients, http://www.doi.org/10.6084/m9.figshare.12651716 ^20^.

### Reporting guidelines

Figshare: STARD checklist for "Diagnostic Accuracy of a Urine Dipstick for Detecting Albuminuria in Hypertensive Patients", http://doi.org/10.6084/m9.figshare.12673154 ^21^.

Data are available under the terms of the Creative Commons Attribution 4.0 International license (CC-BY 4.0).
