# Supplementary material for: Diagnostic accuracy of a urine dipstick for detecting albuminuria in hypertensive patients
Source: F1000Res. 2021 Sep 24;9:1244. Originally published 2020 Oct 15. [Version 3] doi: 10.12688/f1000research.25564.3 (PMC9366238; doi:10.12688/f1000research.25564.3)
Supplement: Peer review history

# Peer review: Reviewer response for version 3

**DOI:** 10.5256/f1000research.77924.r145477

**Published:** 2022-08-10

**Authors:** Jhonatan Mejia

**Affiliations:** 1 Universidad Nacional del Centro del Peru, Huancayo, Peru

The authors aimed to determine the diagnostic accuracy of a urine dipstick for detecting albuminuria in hypertensive patients. They developed a retrospective study in 3067 hypertensive patients from a Thai hospital concluding that a urine dipstick should not be used for mass screening, but a trace result could be used to detect albuminuria.

 The manuscript is well-written, with an adequate design, statistical analysis, and conclusions. Nevertheless, I consider that some minor changes should be considered to enhance the reproducibility and readability.

 Methods

- For further risk of bias evaluation, it will be helpful to clarify if the reference and index test were performed with the same random urine sample. Also, the authors should specify if there was a big gap of time (< 8, ≥ 8 hours) between reference and index test evaluation, along with the preservation method for long intervals.
- It should be literally stated if the test was performed with the analyser or manually, as well as the ACR or albumin cut-off values for “trace, 1+, 2+,3+, and 4+”.

Results

- I believe the term “≥ Negative” is confusing. It would be better to consider “> Negative” and specify the prevalence of albuminuria in another table or only in the text.
- How did the authors obtain a 39.8% albuminuria prevalence? Was ACR ≥ 30 mg/g according to reference test, or ≥ negative cut-off point considered? The authors should clarify this issue.

Is the work clearly and accurately presented and does it cite the current literature?

Yes

If applicable, is the statistical analysis and its interpretation appropriate?

Yes

Are all the source data underlying the results available to ensure full reproducibility?

Partly

Is the study design appropriate and is the work technically sound?

Yes

Are the conclusions drawn adequately supported by the results?

Yes

Are sufficient details of methods and analysis provided to allow replication by others?

Yes

Reviewer Expertise:

NA

I confirm that I have read this submission and believe that I have an appropriate level of expertise to confirm that it is of an acceptable scientific standard, however I have significant reservations, as outlined above.

# Peer review: Reviewer response for version 3

**DOI:** 10.5256/f1000research.77924.r95406

**Published:** 2021-10-06

**Authors:** Surendran Deepanjali

**Affiliations:** 1 Department of Medicine, Jawaharlal Institute of Postgraduate Medical Education and Research, Puducherry, Puducherry, India

The authors have addressed the concerns raised in the previous reviewer's report. However they want to retain the expression  '≥ Negative' even when it is the PPV of the test which is under consideration. The authors state that such a representation is helpful for the reader. Could the authors kindly provide examples of previous studies where the PPV of a negative test is provided?

Is the work clearly and accurately presented and does it cite the current literature?

Partly

If applicable, is the statistical analysis and its interpretation appropriate?

Yes

Are all the source data underlying the results available to ensure full reproducibility?

Partly

Is the study design appropriate and is the work technically sound?

Yes

Are the conclusions drawn adequately supported by the results?

Partly

Are sufficient details of methods and analysis provided to allow replication by others?

Yes

Reviewer Expertise:

urinary tract infections, medical philosophy

I confirm that I have read this submission and believe that I have an appropriate level of expertise to confirm that it is of an acceptable scientific standard, however I have significant reservations, as outlined above.

# Response

**Published:** 2021-10-07

**Authors:** Win Techakehakij

**Affiliations:** Lampang hospital, Thailand

We could not find research in this area presenting the detailed information of diagnostic test parameters, sensitivity, specificity, PPV, and NPV, respecting to each dipstick cutoff, including the Neg. We thus appreciate that presenting this information would be beneficial to the audiences who want to find out in details about this issue.

 Moreover, while it is known that prevalence of the disease in the sample population directly affects PPV, this formation is generally missed in most papers. Providing the PPV information at the Neg cutoff clearly demonstrates the prevalence of proteinuria in the sample and may help audiences gain a better understanding of this research. 

 From the abovementioned benefits, we humbly suggest to keep all diagnostic test parameters for the Neg cutoff in the presentation.

# Peer review: Reviewer response for version 3

**DOI:** 10.5256/f1000research.77924.r95407

**Published:** 2021-10-06

**Authors:** Polathep Vichitkunakorn

**Affiliations:** 1 Department of Family and Preventive Medicine, Faculty of Medicine, Prince of Songkla University, Songkhla, Thailand

This revised manuscript is now adequate for publication.

Is the work clearly and accurately presented and does it cite the current literature?

Yes

If applicable, is the statistical analysis and its interpretation appropriate?

Yes

Are all the source data underlying the results available to ensure full reproducibility?

Partly

Is the study design appropriate and is the work technically sound?

Yes

Are the conclusions drawn adequately supported by the results?

Yes

Are sufficient details of methods and analysis provided to allow replication by others?

Yes

Reviewer Expertise:

NA

I confirm that I have read this submission and believe that I have an appropriate level of expertise to confirm that it is of an acceptable scientific standard.

# Peer review: Reviewer response for version 2

**DOI:** 10.5256/f1000research.58421.r89230

**Published:** 2021-07-16

**Authors:** Surendran Deepanjali

**Affiliations:** 1 Department of Medicine, Jawaharlal Institute of Postgraduate Medical Education and Research, Puducherry, Puducherry, India

The authors have modified the manuscript based on some of the suggestions given in the last review. However, a few points still require further clarification.

1. Abstract: If the authors’ point is that the high PPV is helpful in interpreting the result of an already performed test in that particular study population, then it should be made sufficiently clear in the manuscript. I would like to maintain that discussing the utility of the high PPV of the test after recommending against the use of the test is confusing.
2. Introduction: Reference no.4 is a study comparing 24-hour urinary albumin estimation versus urine spot ACR in patients with diabetic patients. How are the results from this study relevant?
3. Methods: “Laboratory results from the last visit were used if multiple results of a urine dipstick and ACR on the same day were presented within the same patient.” Do the authors mean that the last one of the multiple tests done on the same day was considered for inclusion in the study?
4. The method used to exclude patients with UTI should be mentioned in the manuscript.
5. Results: In my opinion, giving the PPV for ≥ negative test result is not intuitive (in Table 2). This could be modified as > Negative.

Is the work clearly and accurately presented and does it cite the current literature?

Partly

If applicable, is the statistical analysis and its interpretation appropriate?

Yes

Are all the source data underlying the results available to ensure full reproducibility?

Partly

Is the study design appropriate and is the work technically sound?

Yes

Are the conclusions drawn adequately supported by the results?

Partly

Are sufficient details of methods and analysis provided to allow replication by others?

Yes

Reviewer Expertise:

urinary tract infections, medical philosophy

I confirm that I have read this submission and believe that I have an appropriate level of expertise to confirm that it is of an acceptable scientific standard, however I have significant reservations, as outlined above.

# Response

**Published:** 2021-09-23

**Authors:** Win Techakehakij

**Affiliations:** Lampang hospital, Thailand

Abstract: If the authors’ point is that the high PPV is helpful in interpreting the result of an already performed test in that particular study population, then it should be made sufficiently clear in the manuscript. I would like to maintain that discussing the utility of the high PPV of the test after recommending against the use of the test is confusing.

  

- We rechecked throughout the manuscript to ensure emphasis of the Thai population was appropriately addressed.
- As stated in the previous response, implication of the use of sensitivity and PPV information are different. Thus, we briefly provided the key messages of both issues for the audiences.

 

 Introduction: Reference no.4 is a study comparing 24-hour urinary albumin estimation versus urine spot ACR in patients with diabetic patients. How are the results from this study relevant?

  

- We removed the reference no.4.

Methods: “Laboratory results from the last visit were used if multiple results of a urine dipstick and ACR on the same day were presented within the same patient.” Do the authors mean that the last one of the multiple tests done on the same day was considered for inclusion in the study?

  

- It is correct that the last UA results were used in the study if multiple tests were done on the same day. This is as, in reality, there is sometimes errors in performing laboratory test or in reporting results, which require a re-test. Thus, we decidedly chose the last result to minimize these errors.

The method used to exclude patients with UTI should be mentioned in the manuscript.

  

- We added “Patients with the urine results containing white blood cell more than 5-10 cells per high power field were suspected of having urinary tract infections, and thus were excluded from the study” in the method section.

Results: In my opinion, giving the PPV for ≥ negative test result is not intuitive (in Table 2). This could be modified as > Negative.

  

- We suggest to keep the ≥ negative results as providing the results of “≥ negative” is beneficial to audiences to know the prevalence of albuminuria among the study population.

# Peer review: Reviewer response for version 1

**DOI:** 10.5256/f1000research.28212.r86938

**Published:** 2021-06-21

**Authors:** Polathep Vichitkunakorn

**Affiliations:** 1 Department of Family and Preventive Medicine, Faculty of Medicine, Prince of Songkla University, Songkhla, Thailand

The authors examined the diagnostic accuracy of urine dipstick on albuminuria among hypertensive patients in Thailand. This study tried to reveal the sensitivity, specificity, positive predictive value, and negative predictive value of the tool. This is very useful for urine dipstick in many health care settings.

 The manuscript is clear and well documented. The rationale is well established. The authors applied appropriate methods for data analysis and the results were convincing.

 However, to improve paper readability, minor changes are required. I suggest the following:

- The introduction did not discuss the heterogeneity of various accuracy of urine dipstick.
- The authors selected the laboratory results from the last visit. It would be great if you can provide the rationale for this selection method.
- The logic for calculating the sample size is missed in this manuscript. The authors may add the calculation of sample size or power analysis. 
- Table 1: Regarding rounding decimals, the “63.52” should be “63.5”.
- Figure 1: Please explain or discuss the outlier (i.e., ACR ~ 5000+ in the Negative group).
- Table 2: the “95%CI” can be moved to the first row of the table.
- For discussion, the limitations and implications for this research should be mentioned for the readers.

Thank you for the opportunity of reading and evaluating this paper.

Is the work clearly and accurately presented and does it cite the current literature?

Yes

If applicable, is the statistical analysis and its interpretation appropriate?

Yes

Are all the source data underlying the results available to ensure full reproducibility?

Partly

Is the study design appropriate and is the work technically sound?

Yes

Are the conclusions drawn adequately supported by the results?

Yes

Are sufficient details of methods and analysis provided to allow replication by others?

Yes

Reviewer Expertise:

Epidemiology

I confirm that I have read this submission and believe that I have an appropriate level of expertise to confirm that it is of an acceptable scientific standard, however I have significant reservations, as outlined above.

# Response

**Published:** 2021-07-05

**Authors:** Win Techakehakij

**Affiliations:** Lampang hospital, Thailand

The heterogeneity of dipstick results was discussed in details in the discussion.

 In case that there are more than one laboratory results within the same person, we decided to include only one result from each sample to reduce the bias from individuals who had undertaken the tests many times. Results from the last visit were decidedly selected in order to obtain the most recent outcomes in this regard.

 From the Bujang and Adnan (2016), with the prevalence of approximately 40%, the minimum sample size of 408 would yield the conventionally acceptable power of 0.804. However, this study contained a lot more samples of 3,067, which far exceeds the minimum to reassure the accuracy and reliability of the analysis.

 Decimal points were rounded to one digit for percentage and 3 digits for p-value.

 False negative results from dipsticks, including some outliers, ordinarily exist to indicate imperfect predictability of the index test. Possible explanation was described in the discussion part “Likewise, false negative results may have occurred with excessive hydration before collecting the urine specimen, which leads to a decrease in concentration of urine albumin and subsequently a smaller chance of detecting albuminuria.”.

 Owing to the limited space available in the table, we moved the 95%CI to the second line to improve readability for the audiences.

 The main limitation of this research is the externality of the results. This is because the samples were chosen from only one hospital in the northern Thailand, which may not well represent the diagnostic accuracy of the test in other populations, particularly with differential prevalence of albuminuria. This limitation is genuinely the gaps and rationale of this research, which was mentioned in the introduction.

 Concerning the implication, this research mentioned the interpretation and applicability of the results in the discussion part: “results from this study demonstrated that the dipstick has such low sensitivity in detecting albumin in urine. These results suggest that the urine dipstick not be recommended for screening urine albumin in patients with hypertension. In contrast, results of trace or higher yields high PPV, which indicates a very high possibility of the presence of microalbuminuria.”.

 Reference

 Bujang MA, Adnan TH. Requirements for minimum sample size for sensitivity and specificity analysis. Journal of clinical and diagnostic research: JCDR. 2016 Oct;10(10):YE01.

# Peer review: Reviewer response for version 1

**DOI:** 10.5256/f1000research.28212.r85559

**Published:** 2021-06-14

**Authors:** Surendran Deepanjali

**Affiliations:** 1 Department of Medicine, Jawaharlal Institute of Postgraduate Medical Education and Research, Puducherry, Puducherry, India

Panta P *et al* conducted a retrospective study to evaluate the performance of urine dipstick testing for albumin compared to urine albumin-creatinine ratio (ACR) in patients with hypertension. The study population included 3067 subjects of Thailand. The authors found that urine dipstick at the trace positive cut-off has modest sensitivity for detecting ACR of 30mg/g or more. The authors conclude that urine dipstick test cannot be used to screen albuminuria in hypertensive patients.

 Overall the manuscript is well-written. However, there are a set of issues to be addressed in the study.

 Major comments:

**Abstract**

1. Authors state that “there is little evidence” about the diagnostic accuracy of dipstick testing for albuminuria. This information is not accurate since the authors themselves cite studies addressing this issue in the Introduction and Discussion.
2. The conclusion reads” Using the dipstick for screening albuminuria among hypertensive patients should not be recommended due to its low sensitivity. In response to high PPV, a trace threshold of the dipstick may be used to indicate presence of albuminuria.” After having stated that dipstick testing should NOT be recommended, it is redundant to suggest a ‘trace’ threshold for albuminuria.

**Introduction**

1. Although ACR is the ideal test for albuminuria, urine dipstick analysis finds mention as part of initial work up of hypertension in existent guidelines on hypertension (ISH CPG 2020, ESH 2018). Hence in the Introduction authors should elaborate more on the advantages and disadvantages of using the dipstick in comparison to ACR. The references 3-5 do not seem to be appropriate as evidence of recommending ACR for measuring albuminuria.
2. In the last paragraph of Introduction, it is given whether urine dipsticks “confirm” the presence of albuminuria which is not conforming to the ideas in the previous paragraph where it is mentioned as a tool for “screening”.

**Methods**

1. It is mentioned under Participants that “Laboratory results from the last visit were used if multiple results of a urine dipstick and ACR on the same day were presented within the same patient.” It is not clear whether some patients had both the tests repeated on multiple days or multiple results on the same day were available.
2. How did authors exclude patients with UTI?
3. It is not clear whether the urine dipstick reading was done by visual inspection or automated analyzer.  An internet search for EH 2080 reveals it is a urine sediment analyzer. Why was this instrument used for interpreting dipsticks?
4. For calculating BMI and eGFR data on height and weight and serum creatinine were collected. Was it done on the same visit as the urine tests or within any given time frame?

**Results**

1. The authors have not stated what proportion of the study population was detected to have albuminuria in the text. The information has to be deducted from Table 1. Also, it will be more informative if the albuminuria estimated by ACR is quantified into meaningful groups like moderately or severely increased albuminuria. How the dipstick performed in these two groups can also be compared; for example, as given in Reference no.19.
2. It is not clear why statistical tests of significance were used for comparison of 2 ACR groups in Table1. It seems like proportion of diabetic patients with albuminuria is less compared to those without diabetes. The same for CKD also. If the authors want to point out any specific finding through these comparisons it has to be stated in the text. Likewise, comparison of dipstick results between the two groups conveys no extra information.
3. The information provided in Table 2 is not clear. Terms like “≥Negative” and “≥trace” are confusing. A sensitivity of 100% and PPV of 40% for a negative test?

**Discussion**

1. Although the authors have compared their results, especially the low sensitivity of dipstick, with other studies in literature, they have not interpreted their study results thoroughly. The trade-off between the low sensitivity and the good PPV have to be interpreted in terms of population in which the dipstick testing will be employed. According to this study, a trace positive result has 98% PPV in this particular hypertensive population where 70% were diabetic too. The authors could discuss whether the test is still worthy of use from the cost-benefit point of view.
2. If the authors however are maintaining that the test should not be recommended because of low sensitivity, then suggesting a trace threshold for interpretation of test result is superfluous.
3. The ‘variation in calibration’ as a cause for differing diagnostic performance characteristics across populations is not clear enough. Does this apply only to automated analyzer results?
4. The authors state that proteinuria will be over-diagnosed with trace threshold. This point is also not substantiated since a test with a PPV of 98-100% will not have a high false-positivity rate. 

**Conclusion**

1. As mentioned previously in the comments on Abstract, the conclusion has to be re-written without ambiguities. 

**Minor comments**:

1. References 3, 5, 14, 15 & 16 are not correctly cited.
2. The figshare dataset shows age and BMI represented with almost 10 decimal points precision. I would suggest approximating it 2 or 3 decimal points for better representation.
3. The figshare dataset does not give the results of dipstick testing

Is the work clearly and accurately presented and does it cite the current literature?

Partly

If applicable, is the statistical analysis and its interpretation appropriate?

Yes

Are all the source data underlying the results available to ensure full reproducibility?

Partly

Is the study design appropriate and is the work technically sound?

Yes

Are the conclusions drawn adequately supported by the results?

Partly

Are sufficient details of methods and analysis provided to allow replication by others?

Yes

Reviewer Expertise:

urinary tract infections, medical philosophy

I confirm that I have read this submission and believe that I have an appropriate level of expertise to confirm that it is of an acceptable scientific standard, however I have significant reservations, as outlined above.

# Response

**Published:** 2021-07-05

**Authors:** Win Techakehakij

**Affiliations:** Lampang hospital, Thailand

**Abstract**

1. We added “in Thailand” in the text to clarify the scarce of evidence in this area of research.
2. The sentence, “Using the dipstick for screening albuminuria among hypertensive patients should not be recommended due to its low sensitivity”, interprets the implication of the sensitivity of the test, in which it may not be appropriate to apply for mass screening. In this regard, we added “for mass screening” in the text to improve clarity of the content.

On the other hand, the sentence, “In response to high PPV, a trace threshold of the dipstick may be used to indicate presence of albuminuria”, reflects another practical point of using of PPV result when the patients already presenting with results from dipsticks. Unlike the suggestion for mass screening, the PPV suggests that a trace result yields a rather high possibility of the presence of albuminuria and thus may be recommended for use in clinical practice.

**Introduction**

1. The use of ACR as the gold standard for detecting albuminuria is recommended by the Renal Association, with supportive evidence. All the evidence was available in reference 3-5. The pros and cons of using dipsticks in comparison with ACR were discussed in the latter 2 paragraphs with supportive references 6-11.
2. To figure whether urine dipsticks “confirm” the presence of albuminuria does conform the ideas of this paper, which is to explore diagnostic accuracy of the test. Results from diagnostic accuracy can be applied to recommendations for both public screening and clinical diagnosis, as demonstrated in this paper.

**Methods**

1. This means in case of that the patients had multiple results on the same day, as mentioned in the previous sentence, “The study included patients aged 20 and over who were diagnosed with hypertension, ICD10 code “I10-14”, with the results of urine dipstick and ACR from random single spot urine being examined in the same day at least once.”
2. Results of urine dipsticks presenting with red blood cell or white blood cell to the diagnostic criteria of urinary tract infection were construed as urinary tract infection.
3. The urine dipstick reading was done by the automated analyzer. The EH2080 was removed.
4. BMI and eGFR were calculated on the same visit.

**Results**

1. We added “Approximately 39.8% of the samples presented with albuminuria” to describe the proportion of samples with albuminuria.

Concerning the demonstration of albuminuria level, we decidedly omitted this information as this is considered out of the scope of this paper.

1. Statistical analyses shown in Table1 are deemed a compliment to the demonstration of the samples’ characteristics.
2. The terms, e.g., “≥Negative” and “≥trace”, are used when applied the cutoffs for diagnostic test is considered. “≥Trace”, for instance, means that any results with albuminuria higher than the trace level, trace,1+,…,4+, were counted.

A 100% sensitivity when applying the negative threshold is explained by the fact that the negative result is the lowest possible outcome of dipsticks. Using the negative threshold, all the samples, with or without albuminuria, would be identified as having albuminuria, resulting in a 100% sensitivity. 

**Discussion**

1. For the purpose of mass screening in the population level, it may be appropriate to consider only sensitivity, which exhibits the test ability to uncover all diseased patients from the population. Narrative about this was described in the discussion. In addition, issues about high PPV were also provided in the discussion.
2. The issues about difference in interpretation of sensitivity and PPV were above-mentioned.
3. The ‘variation in calibration’ as a cause of the difference in diagnostic performance could apply to not only automated analyzer, but also the visual analyzer.
4. Albuminuria will be over-diagnosed with trace threshold because of PPV of 86.5%. This means that only 865 of 1,000 patients with trace-or-above results will really have albuminuria. Approximately 13.5% will thus be wrongly/overly diagnosed with albuminuria.

**Conclusion**

             Explanation was described above.

**Minor comments:**

1. References were re-checked respecting to the Vancouver style.
2. Information in the dataset was provided with details for the users.
3. Results of the dipsticks were provided with labels.
